# Unconventional electron states in *δ*-doped SmTiO_3_

**DOI:** 10.1038/s41598-017-01847-5

**Published:** 2017-05-08

**Authors:** Frank Lechermann

**Affiliations:** 1 I. Institut für Theoretische Physik, Universität Hamburg, D-20355 Hamburg, Germany; 2 Institut für Keramische Hochleistungswerkstoffe, Technische Universität Hamburg-Harburg, D-21073 Hamburg, Germany

## Abstract

The Mott-insulating distorted perovskite SmTiO_3_, doped with a single SrO layer in a quantum-well architecture is studied by the combination of density functional theory with dynamical mean-field theory. A rich correlated electronic structure in line with recent experimental investigations is revealed by the given realistic many-body approach to a large-unit-cell oxide heterostructure. Coexistence of conducting and Mott-insulating TiO_2_ layers prone to magnetic order gives rise to multi-orbital electronic transport beyond standard Fermi-liquid theory. First hints towards a pseudogap opening due to electron-electron scattering within a background of ferromagnetic and antiferromagnetic fluctuations are detected.

## Introduction

Doped Mott insulators pose a challenging condensed matter problem (see e.g. ref. 1 for a review). At stoichiometry, simple correlated metals show renormalized Landau-like quasiparticles, while charge-gapped Mott (and charge-transfer) insulators often reveal long-range order at low temperature with again a Landau-like order parameter. On the contrary, prominent materials such as e.g. high-T_c_ cuprates, double-exchange driven manganites or the correlated-spin-orbit iridate family prove that doping a Mott insulator can give rise to novel intricate phases, often beyond the Landau paradigm.

The in-depth experimental and theoretical analysis of the effect of random doping in bulk systems is usually hindered by the impact of disorder via the introduced impurities as well as local structural relaxations. This renders the definition of relevant length scales, e.g. screening distances, difficult. Due to the complexity of the problem, many theories of doped correlated materials, especially on the model-Hamiltonian level, neglect details of the local-chemistry aspect. But this may be insufficient to elucidate the subtle energy-scale balancing of strongly correlated electrons systems prone to long-range order.

Two developments are eligible to shed new light on this longstanding problem. First the rising field of oxide heterostructures allows experimentalists to introduce well-defined doping layers in correlated materials^2, 3^. Thereby, the problem of disorder and the ambiguities in identifying unique length scales are removed. Second, the combination of first-principles density functional theory (DFT) with dynamical mean-field theory (DMFT) accounts for the interplay of bandstructure features and many-body effects beyond the realm of static-correlation approaches^4, 5^. Allying these progresses by addressing a doped-Mott-insulator heterostructure via DFT+DMFT is thus suitable to reveal new insight into a hallmark challenge of interacting electron systems.

The distorted perovskite SmTiO_3_ is a member of the *R* TiO_3_ (*R*: rare-earth element) series with formal Ti^3+^ − $3d({t}_{2g}^{1})$ valence configuration and a Mott insulator at stoichiometry. It displays antiferromagnetic (AFM) ordering below *T*_N_ = 45 K. Notably, in the given 3*d* ^1^ titanate series the compound is just at the border of a quantum-critical transition from antiferromagnetic to ferromagnetic (FM) order^6^. Recent experimental work focusing on *δ*-doping SmTiO_3_ with a single SrO layer, exposed non-Fermi-liquid (NFL) character. Moreover, a subtle crossover to still intriguing transport behavior takes place by adding further doping layers^7–9^.

In this work, a realistic many-body approach is employed to resolve the multi-orbital correlated electronic structure of *δ*-doped SmTiO_3_. We reveal a coexistence between itinerant and Mott-insulating regions in real space, associated with different orbital polarizations. Non-Fermi-liquid behavior originates from the internal boundaries between those regions. Eventually, the scattering of itinerant carriers with spin fluctuations near the designed AFM-FM crossover is responsible for a pseudogap fingerprint, giving reason for the realistic NFL regime. These findings pave the way for theoretical investigations of oxide interfaces conducted by materials-design approaches beyond the capabilities of static mean-field studies.

Charge self-consistent DFT+DMFT^10–12^ is used to access the many-body correlated electronic structure, employing a correlated subspace composed of effective (i.e. Wannier-like)^13–16^ Ti 3*d*(${t}_{2g}$) orbitals *w*(${t}_{2g}$) (for more details, see the Supplementary Information). Local Coulomb interactions in Slater-Kanamori form are parametrized by a Hubbard *U* = 5 eV and a Hund’s coupling *J*_*H*_ = 0.64 eV^17^. The multi single-site DMFT impurity problems^18^ are solved by the continuous-time quantum Monte Carlo scheme^19–22^.

## Results

### Mott-insulating SmTiO_3_

**Figure 1:**
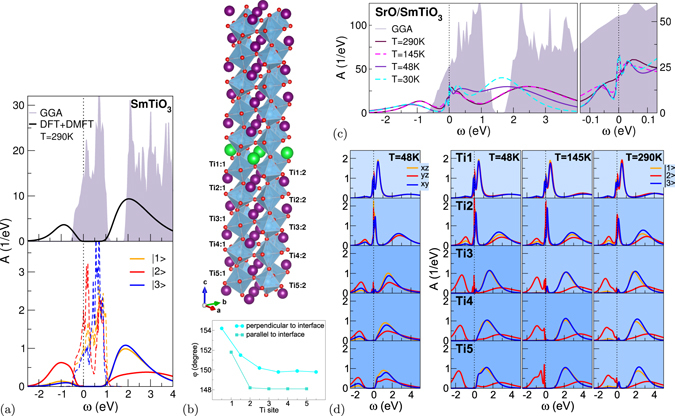
DFT+DMFT results for paramagnetic stoichiometric/*δ*-doped SmTiO_3_. (**a**) Correlated electronic structure of bulk SmTiO_3_. Total (top) and local (bottom) spectral function, with dashed lines for the GGA data. (**b**) Top: supercell of the *δ*-doped compound: Sm (violet), Sr (green), Ti (lightblue), O (small red), bottom: Ti-O-Ti bond angles. (**c,d**) Spectral function of the *δ*-doped system, (**c**) total and (**d**) Ti-resolved. (**d**) Left: for a single temperature in the conventional cubic-harmonic ${t}_{2g}$ basis. Right: over a wider temperature range in the symmetry-adapted Ti-site dependent effective ${t}_{2g}$ basis.

Let us focus first on stoichiometric bulk SmTiO_3_ (cf. Fig. 1a). Besides the lattice parameters, characteristic for the GdFeO_3_-type distorted-perosvskite structure (space group *Pbnm*) are the Ti-O(1,2)-Ti bond angles, whereby O1(2) is the apical(basal in-plane) oxygen position with respect to the *c*-axis^6^. In bulk SmTiO_3_, these angles read *φ*_1_, *φ*_2_ = 146°, 147°. Based on the experimental crystal data^6^, the Ti(${t}_{2g}$) states form an isolated low-energy metallic band manifold of width *W* ~ 1.55 eV in DFT. A small orbital polarization towards an nearly isotropic effective ${t}_{2g}$ state |2〉 = 0.58|*xz*〉 + 0.53|*yz*〉 + 0.62|*xy*〉 is detected. The remaining two effective ${t}_{2g}$ orbitals are given by |1〉 = 0.76|*xz*〉 − 0.63|*yz*〉 − 0.17|*xy*〉 and |3〉 = 0.30|*xz*〉 + 0.57|*yz*〉 − 0.78|*xy*〉. In line with experiment, strong electron correlations drive the material paramagnetic (PM) Mott insulating by effectively localizing a single ${t}_{2g}$ electron on the Ti site. Furthermore, as observed in theoretical assessments of other Mott-insulating 3*d* ^1^ titanates^17, 23, 24^, a substantial orbital polarization, here towards the state |2〉, occurs. The orbital occupation reads (*n*_1_, *n*_2_, *n*_3_) = (0.15, 0.75, 0.10). If we define the charge gap Δ_*g*_ by spectral weight <10^−4^ eV^−1^, a value Δ_*g*_ = 0.55 eV is obtained, in good agreement with the mid-infrared-absorption onset of 0.50 eV^25^. Below its Néel temperature, bulk SmTiO_3_ becomes an G-type antiferromagnet in experiment. Calculations show, that still various magnetic orderings are nearly degenerate in energy (for more details, see the Supplementary Information), in line with the system being on the verge to an AFM-FM transition.

### *δ*-doped SmTiO_3_

#### Lattice structure and correlated electronic structure

In our 100-atom-unit-cell superlattice, *δ*-doping of SmTiO_3_ is established by insertion of a single SrO layer (for more details, see the Supplementary Information). The cell incoporates five symmetry-inequivalent TiO_2_ layers each with two lateral inequivalent Ti sites (see Fig. 1b). The Mott insulator is doped with two holes, i.e. nominally 0.1 hole per Ti site. In the experimental setting of refs 7 and 8, the original *c*-axis is *parallel* to SrO and the original *a*, *b*-axes are inclined. To account for this fact approximatively, we bring the original lattice parameters^6^ in the same directional form, but without lowering the *Pbnm* symmetry, and relax all atomic positions. At the doping layer, the bond angles *φ*_1,2_ are enhanced (see Fig. 1b). There the system is structurally driven towards cubic SrTiO_3_. As expected, beyond 3–4 layers the characteristic angles saturate to bulk-like values^26^. Not surprisingly, this saturation happens faster in terms of layers for the in-plane angle, since the out-of-plane *φ*_1_ is stronger affected from a plane-parallel interface.

The PM many-body electronic structure in the *δ*-doped case is exhibited in Fig. 1c,d. In line with experimental findings^7^, DFT+DMFT reveals metallicity, but with different characteristics as obtained in GGA. The total spectral function *A*(*ω*) shows strong band narrowing and transfer of spectral weight to Hubbard bands. These processes depend rather significantly on the temperature *T*. Already for *T* = 145 K an obvious spectral reduction sets in at low-energy close to the Fermi level. This means that the coherence scale for low-energy excitations is far more lower than in many other correlated bulk systems. Comparison between the *T* = 48 K, 30 K data shows that the overall electronic structure finally settles well below this coherence scale, giving rise to a lower Hubbard band at around −1.2 eV. Notably within a small [−0.1, 0.1] eV energy window around the Fermi level, a three-peak quasiparticle (QP)-like structure emerges. It should not be confused with the conventional large-energy-range three-peak structure involving lower and upper Hubbard bands.

We did not encounter intra- or inter-layer charge-ordering instabilities. It was even impossible to meta-stabilize such charge orderings. Especially the in-plane Ti ions behave equivalent, thus there is no need for intra-layer differentiation in the discussion. However the correlated subspace of *w*(${t}_{2g}$) orbitals becomes layer-TiO_2_ dependent. Still, the Wannier-like functions in the different layers group again in the bulk-established subclasses, and the notion of *w*(${t}_{2g}$) = |1〉, |2〉, |3〉 orbitals in each layer remains coherently applicable.

Different electronic phases are detected with distance to the SrO doping layer (see Fig. 1d). While the nearest TiO_2_ layer is orbital-balanced conducting, the layers beyond the second one are strongly orbital-polarized Mott insulating at low *T*. In between, the second layer is metallic, however it displays strong low-to-high energy spectral-weight transfer and already substantial orbital |2〉 polarization. With rising temperature, the more distant layers partly also become metallic, but in a very incoherent fashion without clear QP formation. The orbital occupations only weakly depend on *T*, but have strong layer dependence (see Table 1). As in the bulk, the layers 3–5 localize one electron in the Ti(${t}_{2g}$) shell, whereas the 2nd layer with about 0.9 electrons is in a doped-Mott state. The first layer with 0.6 electrons appears as a renormalized metal. Hence between different TiO_2_ layers, intricate metal-insulator transitions with strong orbital signature and delicate *T* dependence below room temperature are revealed.

**Table 1:** Temperature-averaged effective Ti(${{\boldsymbol{t}}}_{{2}_{{\boldsymbol{g}}}}$) occupations within each TiO_2_ layer of *δ*-doped SmTiO_3_.

Orbital	Ti1	Ti2	Ti3	Ti4	Ti5
|1〉	0.22	0.29	0.05	0.04	0.04
|2〉	0.16	0.48	0.94	0.95	0.95
|3〉	0.24	0.12	0.01	0.01	0.01
sum	0.62	0.89	1.00	1.00	1.00

#### Transport and magnetism

**Figure 2:**
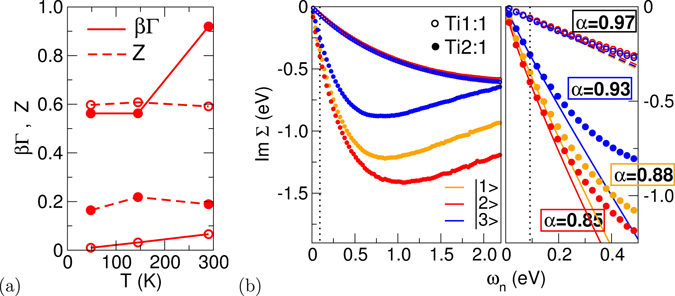
Transport analysis for both conducting layers. (**a**) QP weight and scattering rate for the |2〉 state. (**b**) Orbital-resolved imaginary part of the self-energy on the Matsubara axis *ω*_*n*_ = (2*n* + 1)*πT*. Left: larger frequency range, right: low-frequency region with fitting functions ImΣ(*ω*_*n*_) = *C*_0_ + $A{\omega }_{n}^{\alpha }$ (dashed/full lines). Exponential-fitting cutoff *n*_*c*_ is denoted by the dotted line (for more details, see the Supplementary Information).

For the rest of the paper, we refer to the nearest(next-nearest) TiO_2_ plane with respect to the SrO doping plane as ‘first(second) layer’. In order to assess the transport characteristics of these two conducting layers, the low-frequency behavior of the respective orbital-resolved self-energies Σ(*iω*_*n*_) is analyzed in Fig. 2 (for more details, see the Supplementary Information). *Assuming* an overall Fermi-liquid regime, the QP weight $Z={(1-\frac{\partial {\rm{Im}}{\rm{\Sigma }}(i{\omega }_{n})}{\partial {\omega }_{n}}{|}_{{\omega }_{n}\to {0}^{+}})}^{-1}=\frac{{m}_{{\rm{G}}GA}^{\ast }}{{m}^{\ast }}$ and the electron-electron scattering rate *β*Γ = −*βZ*ImΣ(*i*0^+^) are displayed, whereby m* denotes the effective mass and *β* = 1/*T*. Whereas the first layer indeed shows well-developed Fermi-liquid-like scattering and a moderate *Z*_1_ ~ 0.6, the second layer inherits strong scattering and a much smaller formal *Z*_2_ ~ 0.2. To examine the quality of the Fermi-liquid character, an exponential-function fit is performed to the imaginary part of Σ(*iω*_*n*_), i.e. ${\rm{Im}}\,{\rm{\Sigma }}({\omega }_{n})\mathop{=}\limits^{!}{C}_{0}+A\,{\omega }_{n}^{\alpha }$ ^27^. An ideal exponent *α* = 1 marks a well-defined Fermi liquid with corresponding *T* ^2^-law for the resistivity. Here, indeed Fermi-liquid-like values *α*_1_ = 0.97 and *C*_0_ → 0 are extracted for the first layer, but for the dominant orbital |2〉 in the second layer an exponent *α* = 0.85 and finite intercept *C*_0_ ~ −0.01 eV are obtained. Thus the second layer, mediating between Fermi-liquid and Mott-insulator, is put into a non-Fermi-liquid regime. This happens when the overall coherence scale is already reached, hence a conventional bad-metal picturing is not easily applicable. Note that in experiment, also a subtle NFL regime with *T* ^5/3^-law is measured for *δ*-doped SmTiO_3_^7^.

**Figure 3:**
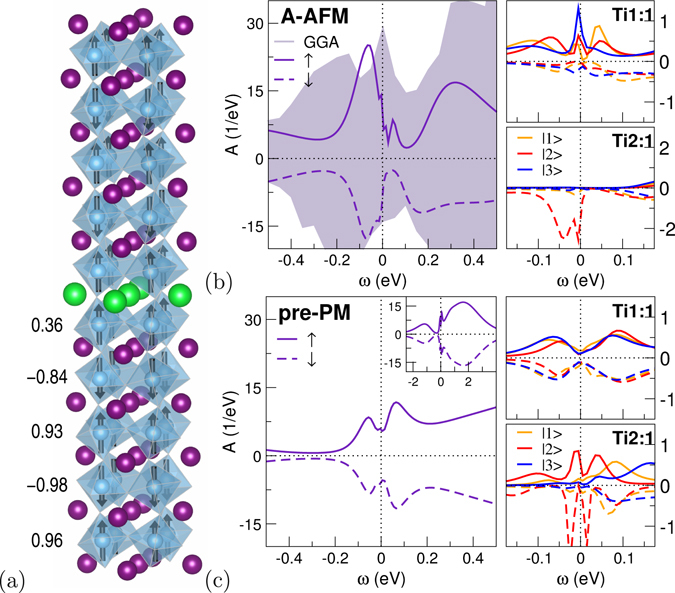
δ-doped SmTiO_3_ with broken spin symmetry (*T* = 48 K). (**a**) A-AFM magnetic order, numbers provide local Ti magnetic moment in *μ*_B_. (**b,c**) Spectral function, left: total, right: local Ti for first and second layer (not shown: similar data for Ti:2 sites). (**b**) A-type AFM phase. (**c**) Pre-converged phase (after 20 DFT+DMFT steps), when starting the self-consistent calculation from the PM solution.

To shed further light onto the nature of the NFL behavior, possible broken-symmetry states are taken into account. Albeit various initializing starting points are investigated, again (spin-broken-assisted) charge-ordering instabilities are not supported by the present theoretical schemes. On the other hand, A-type AFM ordering, i.e. intra-layer FM and inter-layer AFM order, is readily a solution on the GGA level. Starting therefrom, DFT+DMFT quickly converges towards the same-kind many-body A-AFM phase at low temperatures (see Fig. 3a,b). Note that this is not a strict bulk-like A-AFM ordering, but the opposite Ti1 layers sandwiching SrO have identical FM direction with comparatively small magnetic moment. In addition, both Ti5 layers at the respective cell boundary are also FM aligned.

There is strongly reduced total spectral weight at the Fermi level compared to GGA, but the first layer exhibits spin-polarized QP-like peaks at *ε*_F_ associated with an Fermi-liquid-like exponent *α* = 0.95. The delicate second FM layer is again strongly orbital- as well as spin-polarized, and notably already insulating. Intra-layer (or G-type) AFM ordering is not a strong competitor, although various starting points and mixing schemes were applied to stabilize such a metastable solution. Yet the introduced hole doping should indeed weaken the effective (because of strong orbital polarization) half-filled strong-AFM scenario in favor of FM tendencies. Thus part-FM order, especially close to the doping layer and for SmTiO_3_, is not that surprising.

An important observation is made, which delivers information concerning the many-body fluctuations. When starting from the previous PM solution and allowing for spin polarization, DFT+DMFT converges back to the original PM phase via an intriuging intermediate pseudogap state (cf. Fig 3c). The prominent pseudogap signature Δ_P*G*_ ~ 0.1 eV appears after about 15 self-consistency stemps and is quasi-stable for many further calculational steps. Therein, local moments are rather small, i.e. *m*(*Ti*l − 5) = (0.0, −0.02, 0.01, 0.09, 0.04)*μ*_B_. Interestingly, this pseudogap fingerprint in the pre-converged solution is associated with orbital- and spin-balanced electron characteristics in the first layer. The parameters *C*_0_ and *α* are still Fermi-liquid-like in that layer. Yet for Ti2 especially the intercept is rather large with *C*_0_ ~ −0.25 eV. This underlines the strong NFL electron-electron scattering in the second layer.

## Discussion

The electronic states attached to SrO are rather fragile and strongly affected by fluctuations around the PM solution. Due to the dominant magnetic instability in *δ*-doped SmTiO_3_, the pseudogap fingerprint in an intermediate state is interpreted to originate from the proximity to the AFM-to-FM instability. Scattering of first-layer itinerant electrons at emerging moments in the deeper layers below causes a significant spectral-weight reduction at *ε*_F_. Two older perspectives are worth mentioning in this context. Non-Fermi-liquid behavior has been observed in DMFT calculations for a two-orbital Hubbard model due to double-exchange physics induced by orbital selectivity^28^. Seemingly there are some similarities with the present findings, though the two-orbital scenario is here replaced by an effective *two-layer* scenario. In view of the experimental *T* ^5/3^-law for the resistivity, such an exponent is obtained for a metal prone to ferromagnetism^29^ and was e.g. discussed in the context of Ni_3_Al^30^. This may underline the relevance of additional FM fluctuations in the present AFM-based system.

An emerging pseudogap in few-layer SrO-doped SmTiO_3_ is indeed reported in recent tunneling-spectroscopy experiments^31^. Pseudogap behavior is well known for the twodimensional one-band Hubbard model proximate to AFM order^32–36^, described beyond single-site DMFT. But here, intriguing intra/inter-layer-resolved self-energies are sufficient to provide a multi-orbital fingerprint of such a fluctuation-dominated phase in the pre-converged DFT+DMFT cycle. Full stabilization of a pseudogap phase would ask for inter-site self-energies in the theoretical description. However note that the present pseudogap fingerprint does not emerge from sole in-plane correlations *parallel* to the interface, but additionally from perpendicular-to-interface correlations. This may create room for novel designing options of this fluctuation physics in terms of different layering/spacing.

To summarize, we find layer-dependent multi-orbital metal-insulator transitions in *δ*-doped SmTiO_3_ with delicate temperature dependence. Unconventional metallicity for the two TiO_2_ layers close to the SrO doping layer is revealed and nearly completely orbital-polarized Mott-insulating layers beyond. The next-nearest TiO_2_ layer is critical in the sense that it mediates between the Fermi-liquid layer and the Mott layers, resulting in NFL behavior in line with experimental findings. This NFL regime is associated with strong AFM-to-FM spin fluctuations that may lead to a pseudogap structure at the Fermi level. Finally, the present many-body oxide-heterostructure study shall stimulate the investigation of novel emergent electronic phases^37, 38^ by advanced theoretical means on the realistic level.

## Methods

The charge self-consistent DFT+DMFT method is put into practise. For the DFT part, a mixed-basis pseudopotential method^39, 40^, build on norm-conserving pseudopotentials as well as a combined basis of localized functions and plane waves is used. The generalized-gradient approximation (GGA) in the Perdew-Burke-Ernzerhof form ref. 41, is employed. The partially-filled Sm(4*f*) shell is treated in the frozen-core approximation since the highly-localized 4*f* electrons do not have key influence on the present doped-Mott physics. In the mixed-basis, localized functions are included for Ti(3*d*) as well as for O(2*s*) and O(2*p*) to reduce the energy cutoff *E*_*cut*_ for the plane waves.

Our correlated subspace consists of the effective Ti(${t}_{2g}$) Wannier-like functions *w*_*n*_(${t}_{{2}_{g}}$), i.e. is locally threefold. The *w*(${t}_{2g}$) functions are obtained from the projected-local-orbital formalism^13–16^, using as projection functions the linear combinations of atomic ${t}_{2g}$ orbitals, diagonalizing the Ti *w*_*n*_(${t}_{2g}$)-orbital-density matrix. A band manifold of 60 ${t}_{2g}$-dominated Kohn-Sham states at lower energy are used to realize the projection. Local Coulomb interactions in Slater-Kanamori form for the *w*_*n*_(${t}_{2g}$) orbitals are parametrized by a Hubbard *U* = 5 eV and a Hund’s coupling *J*_*H*_ = 0.64 eV. These values for the local Coulomb interactions are common for correlated titanates^17^. The single-site DMFT impurity problems in the supercell are solved by the continuous-time quantum Monte Carlo scheme^19, 20^ as implemented in the TRIQS package^21, 22^. A double-counting correction of fully-localized type^42^ is utilized. To obtain the spectral information, analytical continuation from Matsubara space via the maximum-entropy method is performed. About 40–50 DFT+DMFT iterations (of alternating Kohn-Sham and DMFT impurity steps) are necessary for full convergence.

## Electronic supplementary material

Supplementary Information
