# Supplementary material for: Unconventional electron states in δ-doped SmTiO3
Source: Sci Rep. 2017 May 8;7:1565. doi: 10.1038/s41598-017-01847-5 (PMC5431486; doi:10.1038/s41598-017-01847-5)
Supplement: Supplementary file 1 — Supplementary Information [file 41598_2017_1847_MOESM1_ESM.pdf]

Unconventional electron states in δ-doped $SmTiO_3$

Frank Lechermann

Supplementary Information

MAGNETIC ORDER IN BULK SAMARIUM TITANATE

Bulk $SmTiO_3$ displays antiferromagnetic (AFM) order of G-type form below $T_N = 45\,K$. Yet the compound is just at the border towards ferromagnetism, since already the next rare-earth titanate $R$$TiO_3$ (R: rare-earth element) in the given series, i.e. $GdTiO_3$, is ferromagnetic (FM). To provide insight into the subtle competition between different magnetic orders, we compute the total energies within GGA and GGA+U for the three most prominent AFM orderings, i.e. G-type, A-type and C-type as well as for FM ordering (see Fig. 1). A plane-wave energy cutoff $E_{cut} = 16\,$Ryd and a $9\times9\times9$ k-point grid is applied. The calculations are based on the T=100 K experimental crystal data of Komarek *et al.* [1] and the resulting energies and Ti magnetic moments are displayed in Tab. I.

All ordering cases are at least metastable and the nonmagnetic energy lies always higher, i.e. magnetic ordering is strongly favored at low temperatures. Note however that DFT(+U), in contrast to DFT+DMFT, does not allow to treat the true *paramagnetic* case. For each order, GGA(+U) yields metallic(insulating) solutions. The different ordered states are all very close in energy, at most they differ in about 10(1) meV/atom within GGA(+U). Albeit the numbers designate FM order as most favorable, given the DFT approximation, especially for the Mott-insulating designed GGA+U an energy difference of 1 meV/atom is usually beyond the limit of physical signficance. But the quasi-degeneracy of the magnetic orders is nonetheless physically sound, since as noted, $SmTiO_3$ is just at the AFM-to-FM transition point in the rare-earth titanate series.

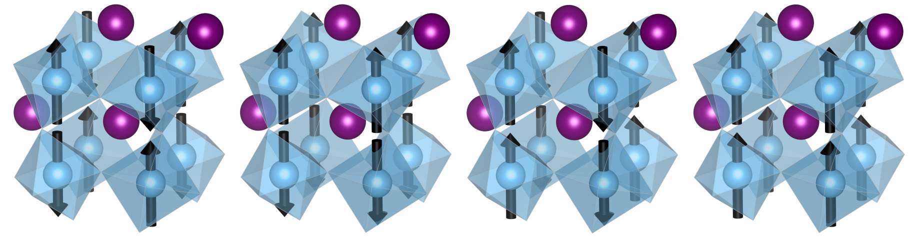

FIG. 1. Competing magnetic orders in $SmTiO_3$.

scheme	G-AFM	A-AFM	C-AFM	FM
GGA	11.3	2.1	10.8	0.0
	0.32	0.62	0.46	0.72
GGA+U	1.2	0.3	1.2	0.0
	0.73	0.75	0.74	0.76

TABLE I. Energy and Ti magnetic moment for different magnetic orders in $SmTiO_3$. First line: energy E_{mag} (in meV/atom) of the magnetically ordered state with respect to the lowest energy in the given set. Second line: local Ti magnetic moment (in μ_B). For GGA+U, the values $U = 5\,eV$ and $J_H = 0.64\,eV$ are chosen.

STRUCTURAL DETAILS FOR THE δ-DOPED ARCHITECTURE

The δ-doped $SmTiO_3$ architecture is realized by a superlattice based on a 100-atom unit-cell. It consists of 10(9) TiO_2(SmO) layers, separated by a single SrO monolayer. Each TiO_2 layer is build from two possibly symmetry-inequivalent Ti ions, to allow for potential intra-layer spin and/or charge ordering. Together with the five-layer resolution distant from the doping layer, there are thus 10 inequivalent Ti single-site DMFT problems in our realistic

modelling. The original lattice parameters [1] are brought in the directional form of the experimental works, i.e. Ref. [2, 3], but without lowering the *Pbnm* symmetry. The original *c*-axis is *parallel* to the doping layer and the original a, b-axes are respectively inclined. The plane-wave energy cutoff is set to $E_{\rm cut} = 11\,$Ryd and a $5 \times 5 \times 3$ k-point grid is used. With fixed lattice parameters, all atomic positions in the supercell are structurally relaxed within DFT(GGA) until the maximum individual atomic force settles below 5 mRyd/a.u.. The lattice distortion introduced by the SrO layer is well captured by the structural relaxations. No relaxation of the lattice paramteres is performed. A change of lattice parameters is expected to be very small due to the structural similarity and does not invoke changes of the key physics discussed in the given work. The obtained crystal structure is used for the PM, A-AFM as well as the pre-converged electronic structure studies.

INFLUENCE OF THE LOCAL-INTERACTION STRENGTH

The chosen local Coulomb interactions are well established for bulk titanates [4]. A first-principles computation of these parameters for the large δ-doped architecture, including their layer dependence, is currently numerically unfeasible. In order to still examine the influence of a smaller/larger local Coulomb interaction, we additionally studied the DFT+DMFT electronic structure for $U = 3.5\,$eV and $U = 6.5\,$eV. The Hund's exchange is even less sensitive and therefore remains fixed at $J_{\rm H} = 0.64\,$eV.

Figure 2 exhibits the layer-dependent Ti orbital filling with U. As expected, for the smaller value of U, the orbital polarization is much weaker for the layers beyond Ti1. Also the Mott state is not reached with distance from the doping layer up to Ti5, thus $U = 3.5\,$eV appears too small to account for the correct δ-doping physics. On the other hand, for $U = 6.5\,$eV the orbital polarization for Ti3-5 is even increased. Moreover, the second TiO_2 layer with Ti2 is

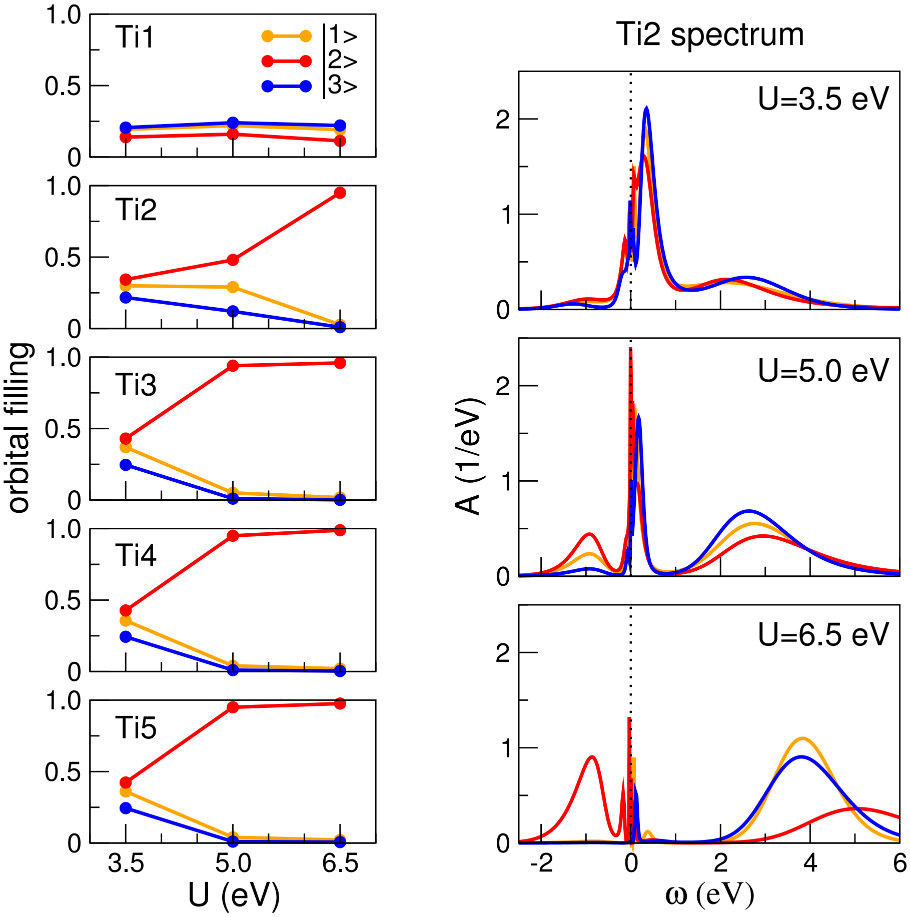

FIG. 2. (color online) Local Ti data for different values of Hubbard U and fixed $J_{\rm H} = 0.64\,$eV ($T = 48$K). Left: layer-dependent paramagnetic orbital filling (with no difference between the intra-layer Ti:1,Ti:2). Right: local Ti2 spectral function with respect to U.

also strongly orbital polarized for this larger U. The additionally shown Ti2 local spectral function in Fig. 2 reveals that the second layer is about to enter a Mott-insulating state. Hence a substantially larger, somewhat unphysical, Hubbard U destroys the revealed two-layer dichotomy.

DETAILS ON THE SELF-ENERGY FITTING

The low-frequency characteristics of the local electronic self-energy within the two TiO_2 layers closest to the SrO doping layer, i.e. Ti1 and Ti2, is analyzed in some detail in the main text. This allows to extract important information on the (non)-Fermi-liquid quality connected to states close to the Fermi energy. To asses this quality, we focus on two features of the self-energy, namely the intercept $C_0 = \lim_{\omega\to 0}\Sigma$ and the form $\tilde{Z}(\omega) = \big(1 - \frac{\partial\Sigma}{\partial\omega}\big|_{\omega\to 0}\big)^{-1}$. For a Fermi liquid (FL) at low temperature, the intercept C_0 approaches zero and $\tilde{Z}(\omega) = Z$ is the constant quasiparticle (QP) weight. Significant deviations from these features signals a non-Fermi liquid (NFL) regime.

We here provide further information on the self-energy fitting procedure in the δ-doped paramagnetic phase to enable such a discrimination, as well as on the grade of those fits. The relevant object in this context is the imaginary part of the Matsubara self-energy. In correct mathematical terms, this function reads $\mathrm{Im}\,\Sigma(i\omega_n)$, with the fermionic Matsubara frequencies defined as $\omega_n = (2n+1)\pi T$. From a sole fitting-function point of view, we however refer in the following to $\mathrm{Im}\,\Sigma(\omega_n)$. Moreover we discuss the fitting parameters as dimensionless, imagining proper normalization.

Figure 3 shows different resolutions of the imaginary part of the multi-orbital self-energy with respect to the frequencies ω_n. The quantum-Monte-Carlo data is well converged at $T = 48\,\mathrm{K}$ to facilitate our low-frequency examination. In order to check the influence on the frequency cutoff n_c (i.e. all frequencies with $n \leq n_c$ are used for the fit), different values for n_c are chosen. Two different stages of fitting procdures are performed and results are here exemplified for the dominant Ti $|2\rangle$ state in the first (Ti1) and second (Ti2) layer next to SrO (see Tab. II). First, motivated by *assuming* Fermi-liquid theory holds, a polynomial fit of simplistic first order ($n_c \leq 4$) and of 5th order ($n_c \geq 6$) is processed. Intercept and Z are therefrom easily extracted from the zeroth and first-order terms, respectively. A good quality of the fitting shall focus on the very low-frequency region, but takes care of the ω_n evolution. Hence the sole linear fitting for very small n_c as well as the higher-order fit for rather large $n_c \sim 16 - 32$ are less suited to the problem. But the results show that a proper fitting leads to robust values for C_0 and Z with e.g. an error $\Delta Z \sim 0.03$.

Questioning Fermi-liquid behavior asks for an exponential fitting function with an exponent α (cf. Tab. II). Only for $\alpha = 1$ a constant QP weight and thus Fermi-liquid behavior is recovered. However the fitting is more exclusively restricted to small n_c. A large n_c results in a too small intercept C_0, since the fitting function tries to account for the natural bending of $\mathrm{Im}\,\Sigma(\omega_n)$ at larger ω_n by shifting C_0 towards zero. Still, the quality of our numerical data is high enough to reveal the resilient FL-to-NFL crossover from the first to the second TiO_2 layer. In the second layer, the exponent α manifestly deviates from unity and the intercept C_0 from zero. Hence electron-electron scattering is beyond FL theory and does not easily vanish at the Fermi energy. Of course the previous FL statements only fully hold for $T \to 0$, but our temperature is already well below a possible QP coherency scale and the contrasting behavior

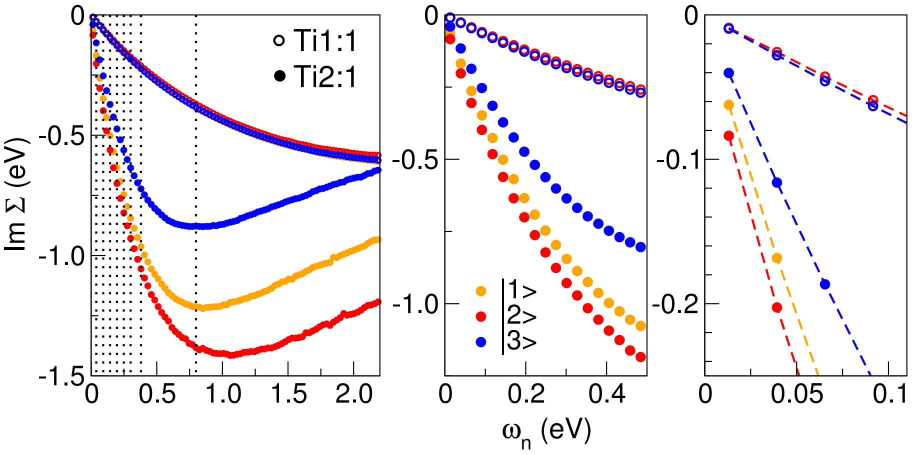

FIG. 3. (color online) Imaginary part of the Ti self-energy on the Matsubara axis for the first two TiO_2 layers with different resolution from left to right. Left: dotted lines mark the frequency cutoff $n_c = 2, 4, 6, 8, 10, 12, 16, 32$ in the different fits (see Tab. II). Dashed lines in the right part are no fitting curves but guides to the eyes.

	$\mathrm{Im}\,\Sigma(\omega_n)=\sum_{k=0}^{1,5} a_k\,\omega_n^k$		$\mathrm{Im}\,\Sigma(\omega_n)=C_0+A\,\omega_n^\alpha$		
n_c	$a_0=C_0$	$(1-a_1)^{-1}$=Z	C_0	A	α
2	-0.0006	0.61	–	–	–
4	-0.0006	0.61	$< 10^{-4}$	-0.61	0.98
6	-0.0016	0.66	$< 10^{-4}$	-0.58	0.96
8	-0.0009	0.63	$< 10^{-4}$	-0.56	0.94
10	-0.0009	0.63	$< 10^{-4}$	-0.54	0.93
12	-0.0004	0.61	$< 10^{-4}$	-0.53	0.92
16	$< 10^{-4}$	0.60	$< 10^{-4}$	-0.51	0.90
32	$< 10^{-4}$	0.60	0.0002	-0.62	0.98
2	-0.0243	0.18	–	–	–
4	-0.0382	0.20	-0.0131	-2.96	0.85
6	-0.0111	0.14	-0.0111	-2.68	0.81
8	-0.0171	0.16	-0.0084	-2.50	0.78
10	-0.0149	0.15	-0.0046	-2.36	0.75
12	-0.0177	0.16	$< 10^{-4}$	-2.25	0.72
16	-0.0189	0.16	$< 10^{-4}$	-2.11	0.69
32	-0.0267	0.17	$< 10^{-4}$	-2.69	0.80

TABLE II. Detailed results for the least-mean-squares fitting procedures applied to $\mathrm{Im}\,\Sigma(\omega_n)$ in the δ-doped case, shown for the Ti $|2\rangle$ state in the first (top) and the second TiO_2 layer (bottom) next to the SrO doping layer. Left columns: polynomial fit, of first order for $n_c = 2-4$ and of fifth order for $n_c = 6-32$. Right colums: exponential fit for $n_c \geq 4$.

in the seemingly FL-like first TiO_2 layer is obvious.

[1] Komarek, A. C. *et al.* Magnetoelastic coupling in RTiO$_3$ (R=La,Nd,Sm,Gd,Y) investigated with diffraction techniques and thermal expansion measurements. *Phys. Rev. B* **75**, 224402 (2007).

[2] Jackson, C. A., Zhang, J. Y., Freeze, C. R. & Stemmer, S. Quantum critical behaviour in confined $SrTiO_3$ quantum wells embedded in antiferromagnetic $SmTiO_3$. *Nat. Commun.* **5**, 4258 (2014).

[3] Mikheev, E., Freeze, C. R., Isaac, B. J., Cain, T. A. & Stemmer, S. Separation of transport lifetimes in $SrTiO_3$-based two-dimensional electron liquids. *Phys. Rev. B* **91**, 165125 (2015).

[4] Pavarini, E. *et al.* Mott transition and suppression of orbital fluctuations in orthorhombic $3d^1$ perovskites. *Phys. Rev. Lett.* **92**, 176403 (2004).
